# Utility of routine ultrasonography follow-up after total thyroidectomy in patients with papillary thyroid carcinoma: a single-center study

**DOI:** 10.1186/s12880-018-0253-9

**Published:** 2018-05-15

**Authors:** Ha Kyoung Park, Dong Wook Kim, Tae Kwun Ha, Young Jin Heo, Jin Wook Baek, Yoo Jin Lee, Young Jun Cho, Dong Kun Lee, Do Hun Kim, Soo Jin Jung, Ki Jung Ahn, Hye Shin Ahn, Hye Jin Baek

**Affiliations:** 1 Department of General Surgery, Busan Paik Hospital, Inje University College of Medicine, Busan, 47392 South Korea; 2 Department of Radiology, Busan Paik Hospital, Inje University College of Medicine, 75, Bokji-ro, Busanjin-gu, Busan, 47392 South Korea; 3 Department of Otorhinolaryngology-Head and Neck Surgery, Dong-A University College of Medicine, Busan, 49201 South Korea; 4 Department of Otorhinolaryngology-Head and Neck Surgery, Busan Paik Hospital, Inje University College of Medicine, Busan, 47392 South Korea; 5 Department of Pathology, Busan Paik Hospital, Inje University College of Medicine, Busan, 47392 South Korea; 6 Department of Radiation Oncology, Busan Paik Hospital, Inje University College of Medicine, Busan, 47392 South Korea; 7 Department of Radiology, Chung-Ang University Hospital, Chung-Ang University College of Medicine, Seoul, 06973 South Korea; 8 Department of Radiology, Gyeongsang National University School of Medicine and Gyeongsang National University Changwon Hospital, Changwon, 51476 South Korea

**Keywords:** Papillary thyroid carcinoma, Total thyroidectomy, Ultrasonography, Follow-up, Recurrence, Surveillance

## Abstract

**Background:**

This study aimed to assess the appropriate number of sessions and interval of routine follow-up ultrasonography (US) in patients who underwent total thyroidectomy for papillary thyroid carcinoma (PTC).

**Methods:**

Between January 2008 and December 2009, 569 patients underwent total thyroidectomy for PTC. Of the 569 patients, 44 were excluded from the study because of no US follow-up data for the neck (*n* = 43) or owing to indeterminate tumor recurrence/persistence (*n* = 1). The follow-up US for all the patients was performed by a single radiologist. Based on the cytohistopathological results, tumor recurrence/persistence was determined.

**Results:**

In the 525 patients, the mean interval to the last follow-up US was 54.7 months, and the mean number of follow-up US sessions was 4.4. Of the 525 patients, 31 (5.9%) showed nodal (*n* = 30) and non-nodal (*n* = 1) tumor recurrence/persistence. Patient age and N stage were independently associated with tumor recurrence/persistence. Among patients showing tumor recurrence/persistence after total thyroidectomy, the time at first detection of suspicious US findings on follow-up US was ≤8 months in 2 patients, between 10 and 23 months in 21, and ≥ 25 months in 8. In a receiver operating characteristic curve analysis, the number of sessions and interval of the provided follow-up US were inappropriate for the detection of tumor recurrence/persistence.

**Conclusions:**

For the detection of tumor recurrence/persistence after total thyroidectomy in PTC patients, routine US follow-up with a 1- or 2-year interval may be excessive.

**Electronic supplementary material:**

The online version of this article (10.1186/s12880-018-0253-9) contains supplementary material, which is available to authorized users.

## Background

Papillary thyroid carcinoma (PTC) is the most common histopathological type of thyroid cancer [1]. However, patients with PTC have a good prognosis because of the indolent nature of the disease [2]. To date, surgical resection is the first-line treatment method for PTC [1]. Although the type of surgery performed depends on multiple factors, such as extrathyroidal extension, nodal metastasis, and patient age, many institutions recommended the use of total thyroidectomy or lobectomy [1, 3, 4]. For avoiding incomplete resection, optimized surgery on the basis of the findings obtained on preoperative imaging, histopathological analysis, and clinico-laboratory analysis is recommended [2]. Nevertheless, tumor recurrence of PTC is common after total thyroidectomy [3, 5–7]. Most cases of tumor recurrence have been found in the regional lymph nodes [2]. For the detection of tumor recurrence in PTC patients, various diagnostic methods including laboratory analysis; whole body scan with radioiodine; and ultrasonography (US), computed tomography, or magnetic resonance imaging have been used.

After surgery, most PTC patients periodically undergo US rather than computed tomography or other imaging modalities to detect locoregional tumor recurrence in the neck [8–10]. US is a commonly used technique for evaluating tumor recurrence because it provides accurate information regarding the lesion size, shape, internal architecture, and vascularity [9, 11]. Unlike computed tomography, US does not involve any radiation hazard. The US features suggestive of metastatic lymph nodes from PTC include nodal enlargement, loss of the fatty hilum, hyperechogenicity, cystic change, microcalcifications, and peripheral vascularity [1]. However, no specific US features have been established for metastatic lymph nodes from PTC. In addition, a diagnosis based on US findings is operator dependent.

Recently, one study demonstrated that an early routine follow-up US in short intervals after lobectomy in patients with papillary thyroid microcarcinoma might be unnecessary [12]. However, the appropriate interval of follow-up US after total thyroidectomy in PTC patients is still unclear. Physicians or institutions use different intervals of routine follow-up US. The unnecessary follow-up US can result in a substantial economic burden to patients. Therefore, this study aimed to assess the appropriate number of sessions and interval of follow-up US in patients who underwent total thyroidectomy for the treatment of PTC.

## Methods

### Patients

Between January 2008 and December 2009, 569 patients underwent total thyroidectomy for the treatment of PTC in our hospital. After total thyroidectomy, patients underwent routine neck US examinations in our hospital, to detect tumor recurrence or nodal metastasis. Of the 569 patients, 43 did not undergo postoperative follow-up US and 1 revealed indeterminate results regarding tumor recurrence/persistence. These patients were excluded from the study. Ultimately, 525 patients (460 women and 65 men, age range, 8–83 years; mean age, 47.6 ± 11.3 years) were included. The institutional review board approved this retrospective study, and the need for informed consent was waived.

### Total thyroidectomy

Total thyroidectomy with a low-collar incision was performed by two independent surgeons with different levels of thyroid surgery experience (29 years and 2 years, respectively). In our hospital, total thyroidectomy was performed for PTC patients with suspicious perithyroidal extension or suspicious nodal metastasis on preoperative imaging including US or computed tomography. Bilateral central nodal dissection was performed in all the cases of total thyroidectomy. The location and size of the primary PTC, perithyroidal extension, nodal metastasis, and multiplicity were investigated on the basis of histopathological results. T and N stages were determined on the basis of the eighth edition of the American Joint Committee on Cancer staging system [13].

### Follow-up neck ultrasonography

A single radiologist (with 6 years of thyroid and neck US experience performing > 4000 US/year) performed the follow-up US examinations by using a high-resolution ultrasound instrument (HDI 5000 or iU 22; Philips Medical Systems, Bothell, WA, USA) equipped with a 5–15-MHz linear probe. In PTC patients in our hospital, postoperative follow-up US in PTC patients is routinely performed at 1- or 2-year intervals. In follow-up US, the following features were investigated on real-time examination: the presence of a newly developed mass in the postoperative thyroid bed and perithyroidal neck area, the presence of a suspicious lymph node in the neck, and US interval changes in the previous mass or in the lymph nodes in the neck. In the literature, suspicious US features of nodal metastasis from PTC include an intranodal cystic component, diffusely increased echogenicity, microcalcification, an irregular margin, and taller-than-wide shape [14, 15].

### Determination of tumor recurrence/persistence

Tumor recurrence/persistence was divided as nodal and non-nodal: nodal tumor recurrence/persistence was defined as the nodal metastasis from PTC on histopathological examinations, whereas non-nodal tumor recurrence/persistence was defined as the presence of PTC in the postoperative thyroid bed or perithyroidal neck area. During the follow-up period after total thyroidectomy, tumor recurrence/persistence was determined by US-guided fine-needle aspiration, core needle biopsy, or surgery.

### Statistical analysis

The normal distribution of continuous data was tested with the Kolmogorov-Smirnov test. Normally distributed variables were compared by using an independent t-test. According to the T stage, N stage, number of follow-up US sessions, and mean intervals of follow-up US, one-way analysis of variance (ANOVA) with the post-hoc Tukey’s test was used for analyzing significant differences among each item. Data are presented as the mean ± standard deviation. Group comparisons of categorical variables were performed by using the Chi-square test and Fisher’s exact test. A receiver operating characteristic (ROC) curve was constructed to determine the intervals and number of follow-up US sessions, with the largest value of area under the ROC curve (Az). A cutoff value for each variable was determined by maximizing the sum of the sensitivity and specificity. The Az values were compared by using the method of DeLong et al. [16]. Multivariate logistic regression analysis was used to investigate the relationships between tumor recurrence/persistence and each item. Statistical significance was accepted as *p* values less than 0.05. All statistical analyses were performed using the IBM SPSS Statistics 19.0 software package (SPSS, Chicago, IL, USA).

## Results

In the 525 included patients, the mean size of the primary PTC was 12.2 ± 8.5 mm (range, 1.8–55.2 mm). The location of the primary PTC was the right lobe in 264 patients, the left lobe in 242, and the isthmus in 19. The T stage was T1a in 278 patients, T1b in 167, T2 in 71, T3a in 7, T3b in 2, T4a in 0, and T4b in 0. The N stage was N0 in 253 patients, N1a in 226, and N1b in 46. Among the 525 patients, there was a solitary PTC in 292 and multiple PTCs in 233. The mean interval from total thyroidectomy to the last follow-up US was 54.7 ± 22.7 months (range, 5–91 months), and the mean number of follow-up US sessions was 4.4 ± 1.8 (range, 1–9).

**Fig. 1:**
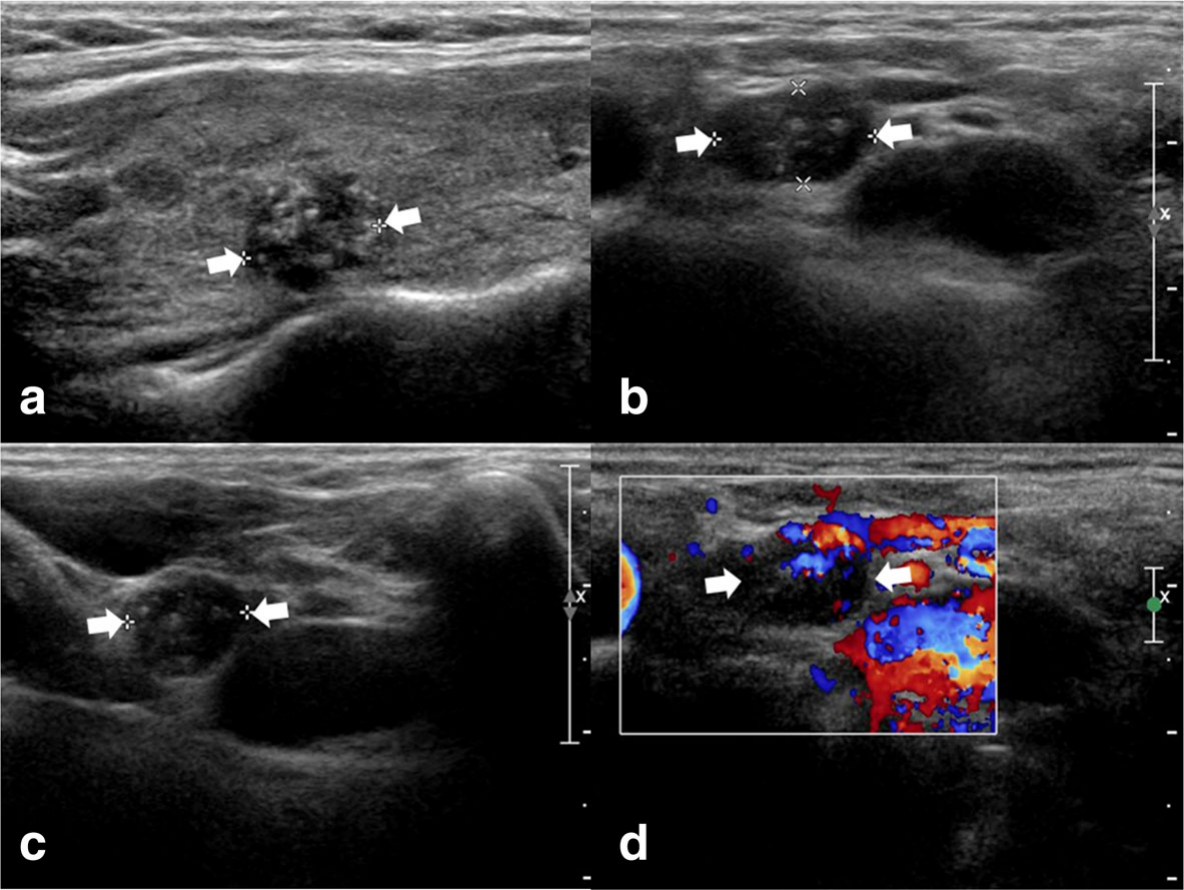
A 45~ 50-year-old patient with tumor recurrence/persistence of papillary thyroid carcinoma (PTC) in a regional lymph node. Before thyroid surgery, a longitudinal gray-scale sonogram (**a**) showed a primary PTC in the right lobe (9.6 mm in the largest diameter). On the 44-month follow-up ultrasonography after total thyroidectomy, transverse (**b**) and longitudinal (**c**) gray-scale sonograms showed a suspicious lymph node with intranodal microcalcifications and diffusely increased echogenicity in the right lower neck (arrows, 6.6 × 8.2 × 11.0 mm). On the transverse color-Doppler sonogram (**d**), a suspicious lymph node showed an increased vascularity. After US-guided fine-needle aspiration for this node, nodal metastasis of PTC was diagnosed on cytology. After consecutive nodal dissection, nodal metastasis was confirmed on histopathological analysis

Of the 525 patients, 31 (5.9%) showed tumor recurrence/persistence: nodal tumor recurrence/persistence in 30 (Fig. 1) and non-nodal in 1. The non-nodal recurrence was found in the postoperative thyroid bed after surgical excision. The final diagnostic method of tumor recurrence/persistence included US-guided fine-needle aspiration in 9 patients and surgical excision in 22. In the tumor recurrence/persistence group, the mean size of the primary PTC was 16.9 ± 9.4 mm (range, 6.4–46 mm). The location of the primary PTC was the right lobe in 20 and the left lobe in 11. The T stage was T1a in 6 patients, T1b in 16, T2 in 8, and T3a in 1. The N stage was N0 in 5 patients, N1a in 18, and N1b in 8. Of the 31 patients with tumor recurrence/persistence, there was a solitary PTC in 13 and multiple PTCs in 18. The mean interval of the last follow-up US was 50.9 ± 26.3 months (range, 10–83 months), and the mean number of follow-up US sessions was 4.3 ± 2.2 (range, 1–9). The mean interval of the follow-up US at the first detection of tumor recurrence/persistence was 19.2 ± 11.7 months (range, 7–50 months).

The data indicating the T stage, N stage, interval of the last follow-up US, and tumor recurrence/persistence are summarized in Table 1. With regard to the T stage, there was no significant difference in the interval of the last follow-up US (*p* = 0.595), whereas there was a significant difference in the tumor recurrence/persistence (*p* = 0.001). For the N stage, there was no significant difference in the interval of the last follow-up US (*p* = 0.096), whereas there was a significant difference in the tumor recurrence/persistence (*p* < 0.0001).

**Table 1:** The T/N stage and tumor recurrence/persistence in 525 patients who underwent total thyroidectomy for papillary thyroid carcinoma

T stage	Interval of the last follow-up US (month: mean ± SD, range)	Tumor recurrence/persistence (%)
T1a (*n* = 278)	53.7 ± 22.9 (5-90)	6 (2.2)
T1b (*n* = 167)	55.4 ± 23.1 (8-91)	16 (9.6)
T2 (*n* = 71)	57.0 ± 20.2 (10-84)	8 (11.3)
T3a (*n* = 7)	64.6 ± 23.5 (14-84)	1 (14.3)
T3b (n = 2)	54 ± 2.8 (52-56)	0
T4a (*n* = 0)	NA	NA
T4b (*n* = 0)	NA	NA
N stage	Interval of the last follow-up US (month: mean ± SD, range)	Tumor recurrence/persistence (%)
N0 (*n* = 253)	53.1 ± 22.9 (5-90)	2.0 (5)
N1a (*n* = 226)	55.1 ± 22.7 (6-91)	8.0 (18)
N1b (*n* = 46)	60.9 ± 21.9 (0-89)	17.4 (8)

Note. *US* ultrasonography, *SD* standard deviation, *NA* not available

The relationship between multiple factors and tumor recurrence/persistence in PTC patients who underwent total thyroidectomy was analyzed by multivariate logistic regression analysis (Table 2). Patient age (odds ratio [OR], 0.96; 95% confidence interval [CI]: 0.93, 0.99) and N stage (OR, 2.49; 95% CI: 1.32, 4.71) were significantly and independently associated with tumor recurrence/persistence, whereas the other factors revealed no significant association. The patients with tumor recurrence/persistence (mean age, 40.9 ± 13.9 years) were younger than those without tumor recurrence/persistence (mean age, 48.0 ± 11.0 years). The tumor recurrence/persistence rate was higher in patients with N1b tumors compared to patients with N1a or N0 tumors. However, there was a significant difference in the initial tumor size between the recurrence and non-recurrence groups according to an independent t-test (*p* = 0.007).

**Table 2:** Multivariate logistic regression analysis of factors predicting tumor recurrence/persistence in 525 patients who underwent total thyroidectomy for papillary thyroid carcinoma

Items	Odds ratio^a^	*P* value
Patient age	0.96 (0.93, 0.98)	0.011
Sex	1.20 (0.38, 3.84)	0.759
Size of primary PTC	1.00 (0.92, 1.10)	0.932
Location of primary PTC	0.56 (0.27, 1.20)	0.136
T stage	1.62 (0.64, 4.10)	0.305
N stage	2.41 (1.27, 4.58)	0.007
Multiplicity	1.88 (0.85, 4.17)	0.119
Interval of the last follow-up US	0.96 (0.92, 1.01)	0.058
Number of follow-up US session	1.49 (0.91, 2.46)	0.116

Note. ^a^Numbers in parentheses are 95% confidence intervals

*PTC* papillary thyroid carcinoma, *US* ultrasonography

The analysis results of follow-up US and tumor recurrence/persistence are listed in Table 3. There was a significant association between the number of follow-up US sessions and the interval of the last follow-up US (*p* = 0.037) and between the number of follow-up US sessions and tumor recurrence/persistence (*p* = 0.042). Of the 31 patients with tumor recurrence/persistence after total thyroidectomy, suspicious tumor recurrence/persistence was detected on follow-up US in ≤8 months in 2 patients, between 10 and 23 months in 21, and ≥ 25 months in 8. In addition, the number of patients with tumor recurrence/persistence was high at the 2nd, 5th, and 6th US follow-up sessions.

**Table 3:** Follow-up ultrasonography and tumor recurrence/persistence in 525 patients who underwent total thyroidectomy for papillary thyroid carcinoma

Number of follow-up US sessions	Interval of the last follow-up US (month: mean ± SD, range)	Tumor recurrence/persistence (%)
1 (*n* = 58)	13.9 ± 11.8 (5-58)	3 (5.2)
2 (*n* = 57)	26.1 ± 14.8 (10-73)	7 (12.3)
3 (*n* = 29)	40.5 ± 13.3 (19-70)	1 (3.4)
4 (*n* = 64)	55.3 ± 11.2 (30-81)	4 (6.3)
5 (*n* = 169)	64.1 ± 9.4 (45-90)	6 (3.6)
6 (*n* = 114)	71.8 ± 6.7 (55-89)	6 (5.3)
7 (*n* = 28)	78.0 ± 9.0 (55-91)	2 (7.1)
8 (*n* = 4)	82.0 ± 0.8 (81-83)	1 (25)
9 (*n* = 2)	82.5 ± 2.1 (81-84)	1 (50)

Note. *US* ultrasonography, *SD* standard deviation

**Fig. 2:**
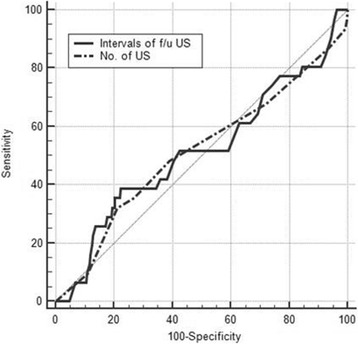
Diagnostic performance of the number of sessions and interval of follow-up ultrasonography for detecting tumor recurrence/persistence after total thyroidectomy in patients with papillary thyroid carcinoma. The diagonal line indicates 50% of the area under the receiver operating characteristic curve and refers to a hypothetical marker that has no power for discriminating a tumor recurrence from a non-recurrence

ROC curve analysis of factors such as the number of sessions and the interval of follow-up US showed no statistical significance in the detection of tumor recurrence/persistence (Table 4). The diagnostic performance related to follow-up US for evaluating tumor recurrence/persistence is shown in Fig. 2. After total thyroidectomy in PTC patients, we found that the number of sessions and the interval of the follow-up US were inappropriate for the detection of tumor recurrence/persistence.

**Table 4:** Multivariate logistic regression analysis of factors predicting tumor recurrence/persistence in 525 patients who underwent total thyroidectomy for papillary thyroid carcinoma

Items	A_z_ value^a^	Sensitivity	Specificity	PPV	NPV	Cut off	*P* value
		(%)	(%)	(%)	(%)		
Number of session	0.513 (0.469, 0.556)	32.3	78.7	8.7	94.9	2	0.828
Interval	0.514 (0.470, 0.558)	38.7	77.5	9.8	95.3	39	0.814

Note. A_z_ indicates the largest area under the receiver operating characteristic curve

*PPV* positive predictive value, *NPV* negative predictive value

^a^Numbers in parentheses are 95% confidence intervals

## Discussion

The recurrence of PTC includes locoregional nodal recurrence, locoregional non-nodal recurrence, and distant metastasis [2]. Nodal recurrence in the neck represents 90% or more of tumor recurrence in postoperative PTC patients [2]. In the present study, most of the tumor recurrence/persistence cases were the locoregional nodal type (96.8%, 30/31) and only 1 case with locoregional non-nodal recurrence/persistence was found. Based on the literature, locoregional recurrence rates after total thyroidectomy in PTC patients range from 5 to 20% [3, 5–7]. The reason for this diversity may be related to the different cohort sizes, subtypes of PTC, different percentage of high risk patients, and follow-up interval [7]. In the present study, the tumor recurrence/persistence rate was low (5.9%) compared to that obtained in the previous studies [3, 5–7]. The tumor recurrence rate may increase when the follow-up interval becomes longer.

In PTC patients, tumor recurrence may be associated with the mortality rate [5]. The decrease or prevention of tumor recurrence is a major concern for most clinicians [17]. The known risk factors for tumor recurrence of PTC include the tumor size, extrathyroidal extension, T stage, and N stage [18, 19]. However, tumor recurrence may be influenced by the number of tumors, follow-up interval, and subtypes of PTC [7]. In the present study, only young age and high N stage were independently associated with tumor recurrence. The reason for discordance in the results is not clear, but it may be associated with the absence of T4 stage cases and the low prevalence of T3 stage tumors (*n* = 9, 1.7%).

For the detection of tumor recurrence after surgery in PTC patients, various methods including laboratory analysis, whole body scan with radioiodine, US, and computed tomography have been used [1]. Unlike serum thyroglobulin measurement or whole-body scans, US can be used to localize a tumor recurrence lesion [10]. Moreover, US can detect tumor recurrences in patients with undetectable serum thyroglobulin levels and negative whole body scan [11]. Thus, postoperative follow-up US has been used widely. Based on the results of the previous study, nodal recurrence in the neck is commonly detected within the first 3-4 years after surgery in PTC patients [20]. In the present study, the majority of nodal recurrence cases were detected on follow-up US within the first 1-2 years (the 1st and 2nd US follow-up sessions). In addition, tumor recurrence/persistence was observed to be high at the 5th and 6th US follow-up sessions, although this might be related to the high prevalence of these session groups (53.9%, 283/525). This result suggests that only 1 or 2 sessions of routine follow-up US within the first 5 years after total thyroidectomy in PTC patients is sufficient for the detection of tumor recurrence/persistence. For example, the first follow-up US can be performed 1-2 years after total thyroidectomy and the second follow-up US can be performed 4-6 years after total thyroidectomy. For confirming these results, a large-scale multi-center study is required.

This study had several limitations. First, all the patients were not included in the study. Of the 569 patients, 44 (7.7%) were excluded because of absent postoperative follow-up US data and indeterminate tumor recurrence/persistence. Second, the frequency and interval of follow-up US were variable. Furthermore, the mean interval of the last follow-up US was 54.7 months, and there were no cases with the last follow-up US > 8 years. Third, no laboratory findings, computed tomography findings, or whole-body scans with radioiodine were included, although postoperative measurements of serum thyroglobulin and thyroid-stimulating hormone levels have been routinely performed in our hospital. However, these findings might influence the detection of tumor recurrence/persistence. Fourth, individual US features, distant metastasis, and pathological subtypes of PTC were not investigated. Finally, we could not distinguish tumor recurrence from persistence.

## Conclusion

This study demonstrated that the tumor recurrence/persistence rate after total thyroidectomy in PTC patients was low, and that the majority of tumor recurrence/persistence cases were detected on follow-up US between 1 and 2 years after total thyroidectomy. Thus, routine US follow-up with a 1- or 2-year interval for the detection of tumor recurrence/persistence may be excessive, and a new algorithm for routine follow-up US should be established.

## Additional file

Additional file 1: Raw data in 525 PTC patients who underwent total thyroidectomy. The data file includes size and location of primary PTC, T/N stage, multiplicity, interval and session number of follow-up US, interval of the last follow-up US, interval between total thyroidectomy and first US detection of tumor recurrence, the presence or absence of tumor recurrence, and the site of tumor recurrence. (XLS 89 kb)

## Abbreviations

CI: Confidence interval
OR: Odds ratio
PTC: Papillary thyroid carcinoma
ROC: Receiver operating characteristic
US: Ultrasonography
